# Aligning attitudes and actions: experience of extreme weather events raises environmental concerns and pro-environmental behaviors of residents

**DOI:** 10.3389/fpubh.2026.1778952

**Published:** 2026-05-08

**Authors:** Shilei Hu, Pengxiang Duan, Yiting Meng, Zhipeng Zhou

**Affiliations:** 1 Harbin Institute of Technology, Weihai, China; 2 Development Research Center of Shandong Provincial People’s Government, Jinan, China

**Keywords:** experience of extreme weather events, media use, private-sphere, pro-environmental behavior, public environmental concern, public-sphere

## Abstract

**Background:**

Under the dual pressures of global climate change and the increasing frequency of extreme weather events, understanding the drivers of public pro-environmental behavior is crucial for achieving sustainable development goals. While existing research has explored various socio-economic and social interaction factors, the role of subjective experiences with extreme weather events and their translation into environmental action remains underexplored. Furthermore, a significant “attitude/intention-behavior gap” persists, and it is unclear whether such experiences foster genuine behavioral change or merely increase environmental concern without corresponding action.

**Methods:**

This study empirically examines the relationship between subjective experiences of extreme weather events and pro-environmental behavior among the Chinese public. Using data from the 2021 Chinese General Social Survey (CGSS), we analyze how severity appraisal of extreme weather events influences both private-sphere and public-sphere pro-environmental behaviors. We employ mediation analysis to test the role of public environmental concern and moderation analysis to assess the influence of media use (traditional vs. new media), while also exploring regional, urban–rural, and environmental self-efficacy-based heterogeneities.

**Results:**

The findings reveal that more severe extreme weather event experiences significantly and positively predict both public- and private-sphere pro-environmental behaviors. Public environmental concern partially mediates this relationship, indicating that disaster experience indirectly promotes action by enhancing environmental awareness. However, new media use negatively moderates the direct effect on private-sphere behavior, weakening this positive influence. Significant heterogeneity exists: residents in Southern China, non-rural areas, and those with high environmental self-efficacy demonstrate a stronger behavioral response to extreme weather events compared to their counterparts in Northern China, rural areas, and those with low self-efficacy.

**Conclusion:**

Extreme weather experiences can serve as a catalyst for pro-environmental action, effectively bridging the gap between environmental concern and behavior. To leverage this potential, policymakers should integrate behavioral promotion into post-disaster recovery, strengthen environmental risk communication, and optimize new media content to provide clear, actionable guidance rather than sensationalized or fragmented information. Furthermore, differentiated strategies are needed to support less responsive groups through enhanced infrastructure, community-based training, and psychological empowerment programs, thereby fostering a more inclusive and effective pathway to climate-resilient sustainable development.

## 1 Introduction

In recent years, the intensification of global climate change and the increasing frequency of climate disasters have posed severe challenges to human survival and development (1). According to the Sixth Assessment Report (AR6) of the Intergovernmental Panel on Climate Change (IPCC), the frequency and intensity of climate disasters are likely to increase further over the next few decades, posing a significant challenge to the achievement of global sustainable development goals (SDGs). Extreme weather events such as heatwaves, droughts, heavy rains, and floods not only directly threaten human life and property (2), but also cause serious damage to the ecological environment. These disasters, largely related to human activities, can be effectively mitigated through pro-environmental behavior, which has become an important force in combating climate change and achieving sustainable development.

Pro-environmental behavior, also known as environmental protection behavior or eco-friendly behavior, refers to actions that can reduce environmental harm and improve environmental quality (3). As pro-environmental behavior has gained increasing attention, many recent studies have focused on its drivers among the public. However, these studies have mainly examined the effects of social interaction, internet use, peer effects, as well as the public’s education level and economic status (4–8). Some recent research has looked at the impact of extreme weather events on residents’ pro-environmental behavior, but these studies focus on objective weather events rather than individuals’ subjective evaluation of experiencing extreme weather events in residential areas. Indeed, studies show that people only tend to act pro-environmentally when extreme weather hits their own area, whereas impacts on neighboring areas are limited by psychological distance, an “only in my backyard” phenomenon (9). Hence, findings based on regional extreme weather events data may differ from those derived from individual-level disaster experiences. This partially explains why recent studies have found that extreme weather events do not translate into pro-environmental behavior (10); instead, they may even inhibit it (11, 12).

Meanwhile, existing research has identified a “attitude/intention-behavior gap” in the public’s pro-environmental behavior (13). This raises an important question: does experience with extreme weather events simultaneously increase both environmental concern and pro-environmental behavior, or does it only affect the former (words) rather than the latter (deeds)? Recent studies have reached inconsistent conclusions (10, 14–16). This calls for new empirical evidence to further clarify the relationship between extreme weather events and the public’s environmental concern and pro-environmental behavior.

Against this backdrop, this study aims to explore the relationship between experience with extreme weather events and public pro-environmental behavior, and to further reveal the underlying mechanisms, so as to provide theoretical support and policy recommendations for promoting public participation in environmental protection. The marginal contributions of this paper are threefold. First, this study simultaneously focuses on the effects of extreme weather events on residents’ environmental concern and pro-environmental behavior (including private-sphere and public-sphere behaviors), providing new evidence on whether the public’s words and deeds are consistent in the context of extreme weather events. Second, unlike existing studies that focus on the impact of extreme weather events per se, this study explores the effect of subjective experience of extreme weather events (i.e., event severity appraisal) on public pro-environmental behavior. Third, using data from the Chinese context, this study reveals the mechanisms through which experience with extreme weather events influence public pro-environmental behavior, as well as the unique role of social media in moderating the relationship between the two.

## 2 Theoretical analysis and research hypotheses

### 2.1 Experience of extreme weather events and public pro-environmental behavior

We posit that experiencing extreme weather events significantly alters public pro-environmental behavior in both the private and public spheres. Pro-environmental behaviors in the private sphere refers to individual, autonomous actions characterized by low social visibility and direct, measurable micro-scale impacts (17). These behaviors are primarily driven by personal norms, cost–benefit evaluations, and habitual reinforcement (17). Examples include saving water and electricity, reducing the use of disposable household items, recycling recyclable materials, and purchasing green products (18, 19). Pro-environmental behaviors in the public sphere, in contrast, are collective actions aimed at influencing socio-political or infrastructural systems to achieve environmental goals (17). These involve public institutions and communities, such as advocating for pro-environmental behavior in the public domain or urging energy conservation and environmental protection in public places (19, 20).

People often perceive climate change as a distant problem, temporally in the future, spatially elsewhere, and socially relevant to others (21). But a flood, heatwave, or typhoon that strikes their own community instantly pulls the risk to a zero-distance. When people personally experience weather anomalies, the psychological distance of climate change (i.e., the sense that it is remote in time, space, and social terms) may be reduced. As this psychological distance shortens, people are more likely to see climate change as an urgent priority and to weaken belief in climate change conspiracy theories (22). After experiencing a weather-related disaster, people often reflect, “What can I do to avoid being harmed next time?” This reflection points in two directions. On the one hand, to cope with future risks, individuals adopt private-sphere pro-environmental behaviors, actions that are relatively low-cost, highly controllable, and provide immediate psychological comfort of “doing something.” On the other hand, when they realize that individual efforts alone cannot solve climate problems, they turn to collective and policy-based protection (18, 23). At this point, engaging in public climate response actions becomes a rational act of “buying collective insurance.” In summary, experience with extreme weather events stimulates both self-rescue behaviors at the individual level and demands for other-rescue or systemic change at the public level, thereby enhancing public pro-environmental behavior in both the private and public spheres. Based on the above analysis, we propose the following hypotheses:

*H1a*: The more severe the experience of extreme weather, the more likely the public is to engage in public-sphere pro-environmental behavior.

*H1b*: The more severe the experience of extreme weather, the more likely the public is to engage in private-sphere pro-environmental behavior.

### 2.2 The mediating effect of public environmental concern

Personally experiencing extreme weather events makes the abstract concept of climate change instantly tangible, visceral, and frightening (24). This experience directly engages people’s risk perception system, prompting them to reassess the severity, personal relevance, and temporal urgency of environmental problems (25, 26). Existing research show that experiencing extreme weather sounds a psychological alarm, raising the cognitive and emotional salience of environmental issues (27). Meanwhile, according to norm activation theory, disaster experiences can activate intrinsic norms, enhance public awareness and concern, and foster a sense of responsibility to protect the environment (28). Hence, extreme weather experiences increase public environmental concern.

Heightened environmental concern leads people to seek solutions in both the private and public spheres. On the one hand, environmental concern strengthens the intrinsic motivation of “what can I do?” thereby promoting private-sphere behaviors. On the other hand, it also exposes the fragility of existing infrastructure and emergency response systems, transforming personal worry into political and social action demanding systemic solutions (i.e., public-sphere behaviors). Thus, public environmental concern mediates the relationship between extreme weather event experiences and public pro-environmental behavior (in both public and private spheres), forming the psychological process of “external shock-attitude reconstruction-behavioral transformation.” Existing studies has also shown that environmental concern plays a mediating role between extreme weather events and public adaptive behavior (27). Without this mediator, extreme weather might leave only traumatic memories rather than cultivating genuine environmental action. This also explains why a few studies have found that experiencing extreme weather events can influence environmental concern but fails to change behavior (16). The reason may be that recovery and reconstruction costs after severe trauma constrain the available budget for environmental protection (11, 29). Based on the above analysis, we propose the following hypotheses:

*H2a*: Public environmental concern plays a mediating role between experience with extreme weather events and the public’s public-sphere pro-environmental behavior.

*H2b*: Public environmental concern plays a mediating role between experience with extreme weather events and the public’s private-sphere pro-environmental behavior.

### 2.3 The moderating effect of media use

Media play an important role in shaping public environmental cognition and behavior (30). Traditional media mainly refer to authoritative outlets such as television, newspapers, and radio (31). They play a moderating role in the relationship between extreme weather events and pro-environmental behavior primarily by shaping public risk perception and responsibility attribution. On the one hand, traditional media act as “risk amplifiers”: they increase public attention to ecological issues (32), maintain the perceived importance of climate topics through extensive coverage, and elevate scattered personal experiences (e.g., my city is flooded) into public issues (e.g., climate change is real). On the other hand, the authoritative interpretation of traditional media can shift public internal versus external attribution of extreme weather events (33). If coverage clearly links disasters to climate change, it effectively enhances public awareness of internal attribution (i.e., the belief that events are related to human actions), thereby strengthening the sense of responsibility to solve the problem.

New media mainly refer to social media, short-video platforms, and the like (31). They play a moderating role in the relationship between extreme weather events and pro-environmental behavior primarily by facilitating the flow of information and emotions, as well as shaping social norms. Extreme weather prompts people to post relevant information more frequently on social media (34). This real-time sharing quickly generates emotional resonance (35). Meanwhile, when people frequently see others sharing pro-environmental behaviors (e.g., using reusable bags, sorting waste) and discussing climate change, they come to realize that “everyone is acting.” This perceived social norm, as a behavioral reference, significantly catalyzes their own participation (36, 37). Based on the above analysis, we propose the following hypotheses:

*H3a*: New media use positively moderate the relationship between experience with extreme weather events and the public’s public-sphere pro-environmental behavior.

*H3b*: New media use positively moderate the relationship between experience with extreme weather events and the public’s private-sphere pro-environmental behavior.

*H4a*: Traditional media use positively moderate the relationship between experience with extreme weather events and the public’s public-sphere pro-environmental behavior.

*H4b*: Traditional media use positively moderate the relationship between experience with extreme weather events and the public’s private-sphere pro-environmental behavior.

**Figure 1:**
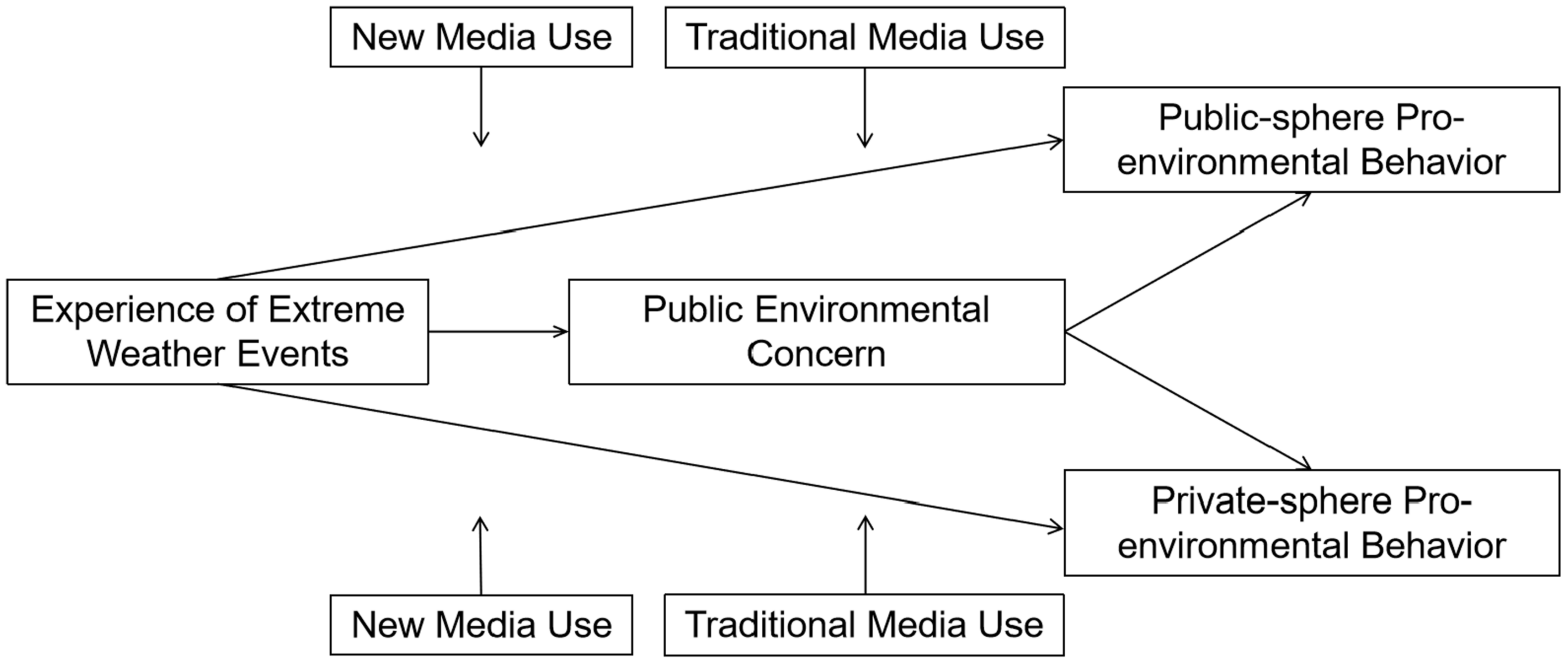
Conceptual model.

The conceptual model diagram is shown in Figure 1.

## 3 Research design

### 3.1 Data source

The data used in this study are from the Chinese General Social Survey (CGSS) 2021. Initiated in 2003, the CGSS is China’s first national, comprehensive, and ongoing academic survey. The 2021 wave included an environmental module from the International Social Survey Programme (ISSP), covering topics such as climate change, environmental pollution hazards, and environmental protection behaviors. After excluding cases with missing variables, 1,929 observations were retained for analysis.

### 3.2 Variable description

The dependent variables in this study are public pro-environmental behaviors, including both public-sphere and private-sphere pro-environmental behaviors.

Following prior studies (38, 39), public-sphere pro-environmental behavior was measured by asking respondents whether they had engaged in any of the following four actions: joining an environmental organization, signing a petition on environmental issues, donating to an environmental organization, or participating in a protest or demonstration for environmental causes. Respondents who engaged in at least one of these behaviors were coded as 1, and those who did not as 0.

Drawing on relevant studies (38, 39), private-sphere pro-environmental behavior was measured by asking respondents how often (1 = never; 2 = sometimes; 3 = often; 4 = always) they engaged in two behaviors: (1) recycling glass, aluminum cans, plastics, and newspapers, and (2) avoiding purchasing certain products for environmental reasons. Responses were averaged across these behaviors, with higher scores indicating more frequent pro-environmental behavior.

The independent variable is the experience of extreme weather events. Drawing on previous studies (25, 40, 41), this study uses “severity of extreme weather events” as the independent variable. In the 2021 CGSS questionnaire, respondents were asked: “How severe do you think extreme weather events are in your residential area? (1 = not severe; 2 = not very severe; 3 = moderate; 4 = somewhat severe; 5 = very severe)” Assessing the severity of extreme weather events in one’s residential area helps capture the impacts and losses caused by climate disasters, thereby providing solid data support for measuring extreme weather experiences.

Public environmental concern serves as the mediating variable in this study. Respondents were asked: “Overall, how concerned are you about environmental issues?” Responses were recorded on a five-point scale (1 = not concerned at all, 2 = not very concerned, 3 = neutral, 4 = somewhat concerned, 5 = very concerned), effectively quantifying the public’s subjective level of environmental concern. This measurement approach has been widely adopted in previous studies (42, 43).

Media use serves as the moderating variable in this study, encompassing both traditional and new media use. In the questionnaire, respondents’ frequency of using different media (newspapers, magazines, radio, television, the Internet (including mobile phone Internet access), and mobile customized messages) was measured on a 5-point scale (1 = never, 2 = rarely, 3 = sometimes, 4 = often, 5 = always). This question has been widely used to measure media use in previous studies [e.g., (31, 44)]. The reliability test yielded a Cronbach’s alpha of 0.615, indicating acceptable internal consistency for factor analysis. Principal component analysis (PCA) extracted two factors: the “traditional media factor” (newspapers, magazines, radio, television) and the “new media factor” (Internet and mobile customized messages) (Table 1), thereby enabling the study to divide media use into traditional and new dimensions. Traditional media use, represented by newspapers, magazines, radio, and television, constitutes the cornerstone of pre-Internet mass communication and helps assess individuals’ information contact patterns in the traditional media environment (31). New media use, measured by Internet and mobile phone customized messages, captures both active and passive information acquisition modes (31).

**Table 1:** Principal component analysis of media use.

Item	Traditional media factor	New media factor
1. Newspapers	0.428	−0.005
2. Magazines	0.400	0.108
3. Radio	0.338	−0.099
4. Television	0.257	−0.390
5. Internet (including mobile phone Internet access)	−0.023	0.551
6. Mobile customized messages	0.058	0.449

To comprehensively analyze the factors influencing the public’s pro-environmental behavior, we drew on relevant studies (4–7) and introduced a range of control variables. These variables cover basic demographics and socioeconomic backgrounds, including respondents’ age, gender, marital status, educational level, religious belief, political affiliation, income level, household registration status (*hukou*), socioeconomic status, and family economic status.

Specifically, age refers to the respondent’s actual age. For gender, male is coded as 1 and female as 0. For marital status, married is coded as 1 and unmarried as 0. Education level is measured by years of schooling: illiterate = 0; primary school and private school = 6; junior high school = 9; senior high school (including vocational high school), secondary technical school, and technical school = 12; associate degree (including adult higher education) = 15; bachelor’s degree (including adult higher education) = 16; postgraduate and above = 19. For religious belief, those with religious belief are coded as 1, and those without as 0. For political affiliation, Communist Party of China (CPC) members are coded as 1, and others as 0. Income level is measured as the natural logarithm of last year’s annual income plus one. For household registration (hukou), non-agricultural hukou is coded as 1, and agricultural hukou as 0. Socioeconomic status is measured by the survey question: “Overall, where do you think your own socioeconomic status stands in society?” (Lower class = 1; Lower-middle class = 2; Middle class = 3; Upper-middle class = 4; Upper class = 5). Family economic status is measured by the survey question: “How does your family’s economic status compare to the average in your local area?” (Far below average = 1; Below average = 2; Average = 3; Above average = 4; Far above average = 5).

In addition, environmental self-efficacy was included as a control variable (30, 45), measured by the statement: “My efforts to protect the environment are meaningless unless everyone else does the same.” To account for individual heterogeneity, provincial dummy variables were also introduced. Descriptive statistics for these variables are presented in Table 2.

**Table 2:** Descriptive statistical analysis.

Variables types	Variables	Mean	SD	Min	Max
Dependent variable	Pro-environmental behavior in the public sphere	0.15	0.357	0	1
	Pro-environmental behavior in the private sphere	2.273	0.827	1	4
Independent variable	Experience with extreme Weather events	2.671	1.215	1	5
Mediating variable	Environmental concern	3.59	0.914	1	5
Moderating variables	Traditional media use	2.893	0.956	1.366	7.29
	New media use	1.514	1.438	−1.362	4.825
Control variables	Age	49.863	17.437	18	94
	Gender	0.466	0.499	0	1
	Marital status	0.779	0.415	0	1
	Education level	9.749	4.618	0	19
	Religion belief	0.031	0.174	0	1
	Political affiliation	0.127	0.333	0	1
	Income level	9.12	4.611	0	16.118
	Household registration status (Hukou)	0.426	0.495	0	1
	Socioeconomic status	2.267	0.888	1	5
	Family economic status	2.601	0.779	1	5
	Environmental self-efficacy	2.612	1.186	1	5

### 3.3 Model specification

To examine the impact of extreme weather event experiences on the public’s pro-environmental behavior, two regression models can be designed: one for public-sphere pro-environmental behavior and the other for private-sphere pro-environmental behavior. Given that public-sphere pro-environmental behavior is a binary variable (0–1), it is suitable for a logistic regression model, whereas private-sphere pro-environmental behavior is a continuous variable, making it suitable for a linear regression model.

## 4 Empirical analysis results

### 4.1 Descriptive statistics analysis

**Figure 2:**
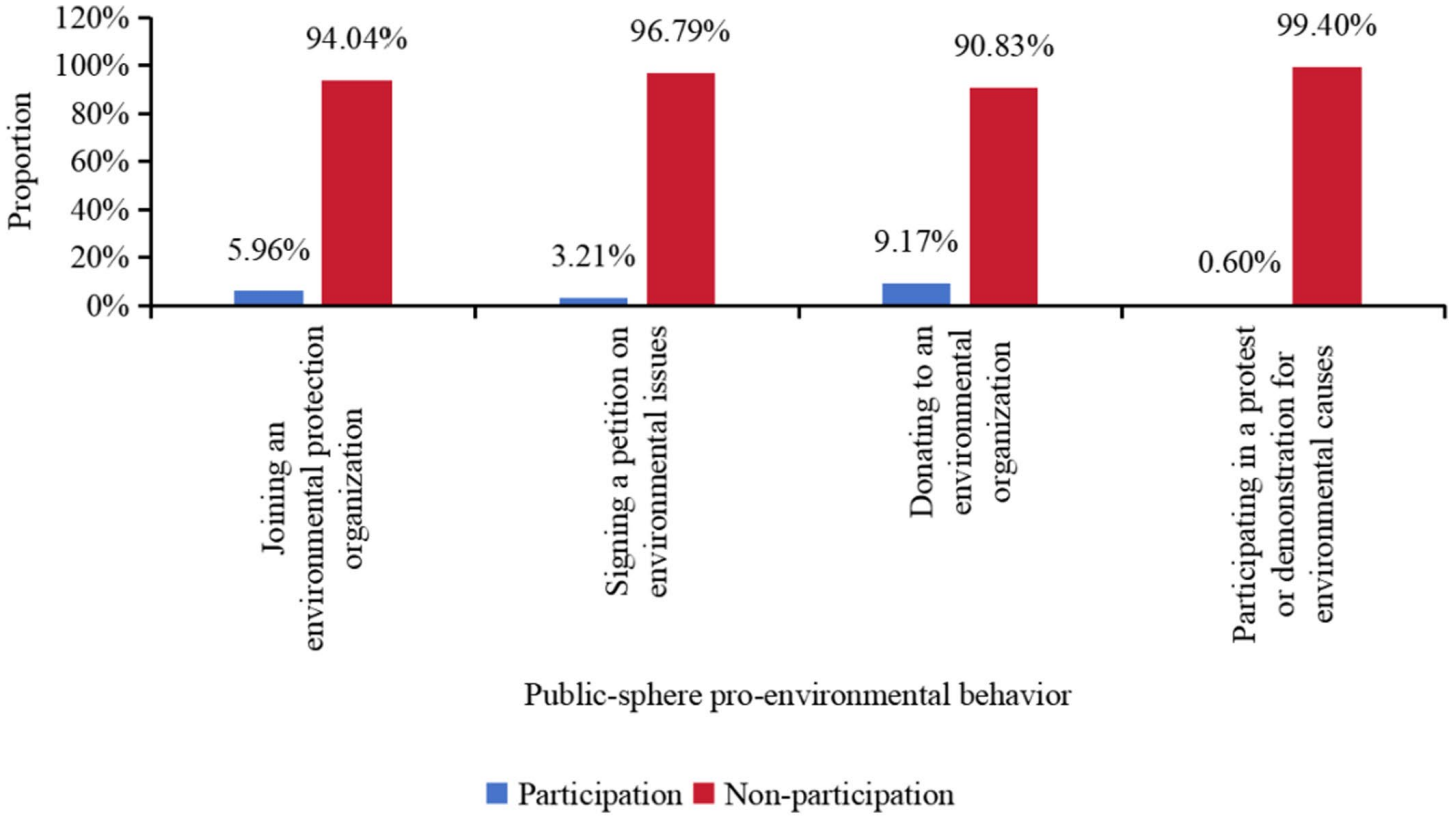
Distribution of public-sphere pro-environmental behavior.

Figure 2 illustrates the characteristics of the public’s public-sphere pro-environmental behavior. Specifically, the participation rates for joining environmental protection organizations, signing environmental petitions, donating money to environmental groups, and participating in environmental protests or demonstrations were 5.96, 3.21, 9.17, and 0.60%, respectively. These findings indicate that overall participation in public-sphere pro-environmental behaviors is relatively low. Notably, the participation rate in protests or demonstrations is particularly low (only 0.60%), likely due to the high costs and potential risks associated with these actions. In contrast, the participation rate for donating money to environmental groups is relatively higher (9.17%), though still below 10%, suggesting limited public enthusiasm for economic support.

**Figure 3:**
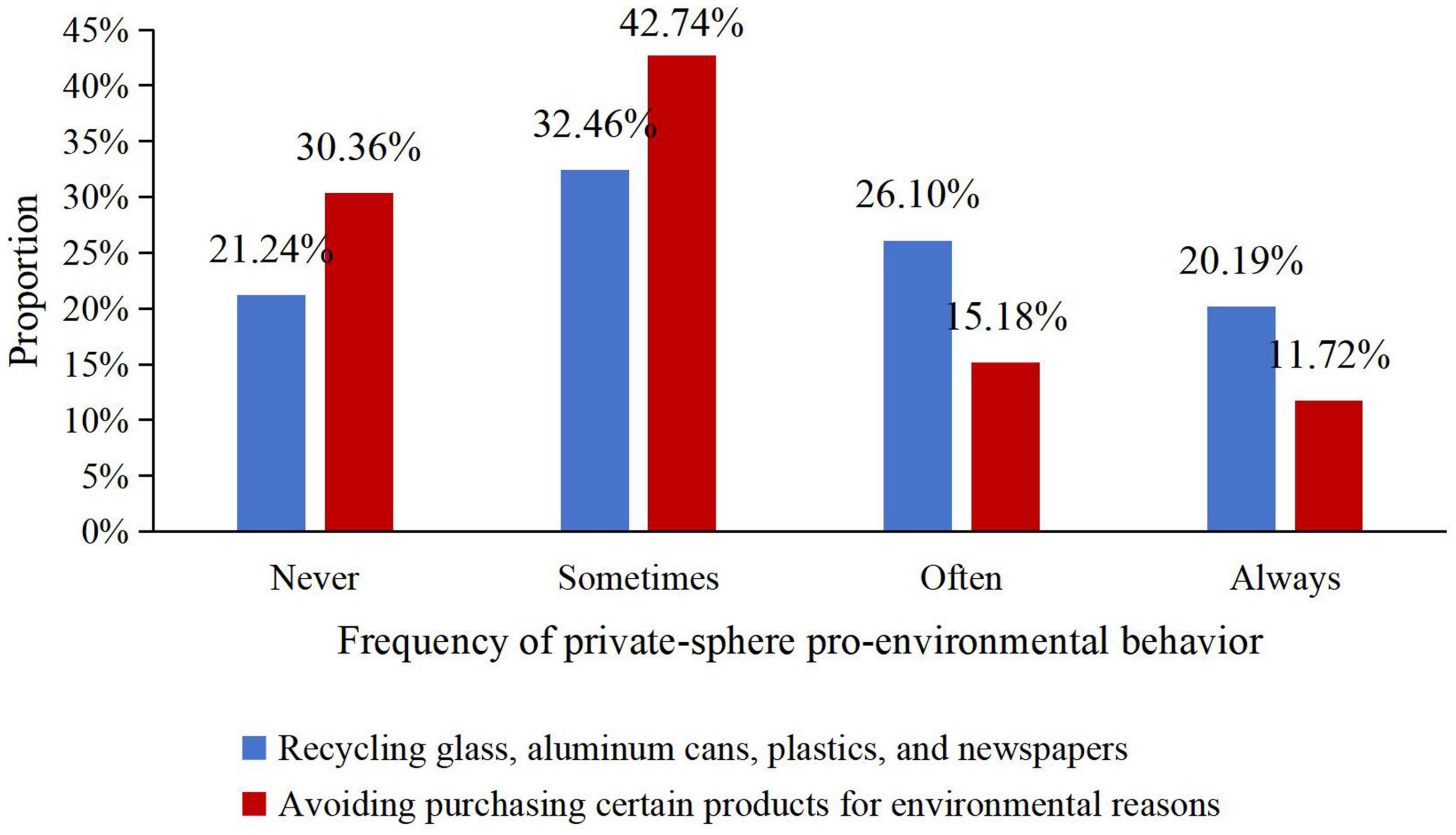
Distribution of private-sphere pro-environmental behavior.

In the classification statistics of private-sphere pro-environmental behavior, the participation rate in waste sorting (glass, aluminum cans, plastic, and newspapers) shows a certain hierarchy. Specifically, as shown in Figure 3, 20.19% of respondents reported “always sorting,” while 21.24% reported “never sorting,” indicating that the public’s implementation of waste sorting still needs improvement. Additionally, regarding the behavior of avoiding purchasing certain products for environmental reasons, the combined proportion of “never” and “sometimes” is as high as 73.10%, indicating that public awareness and behavior in the private sphere have not yet become mainstream.

**Figure 4:**
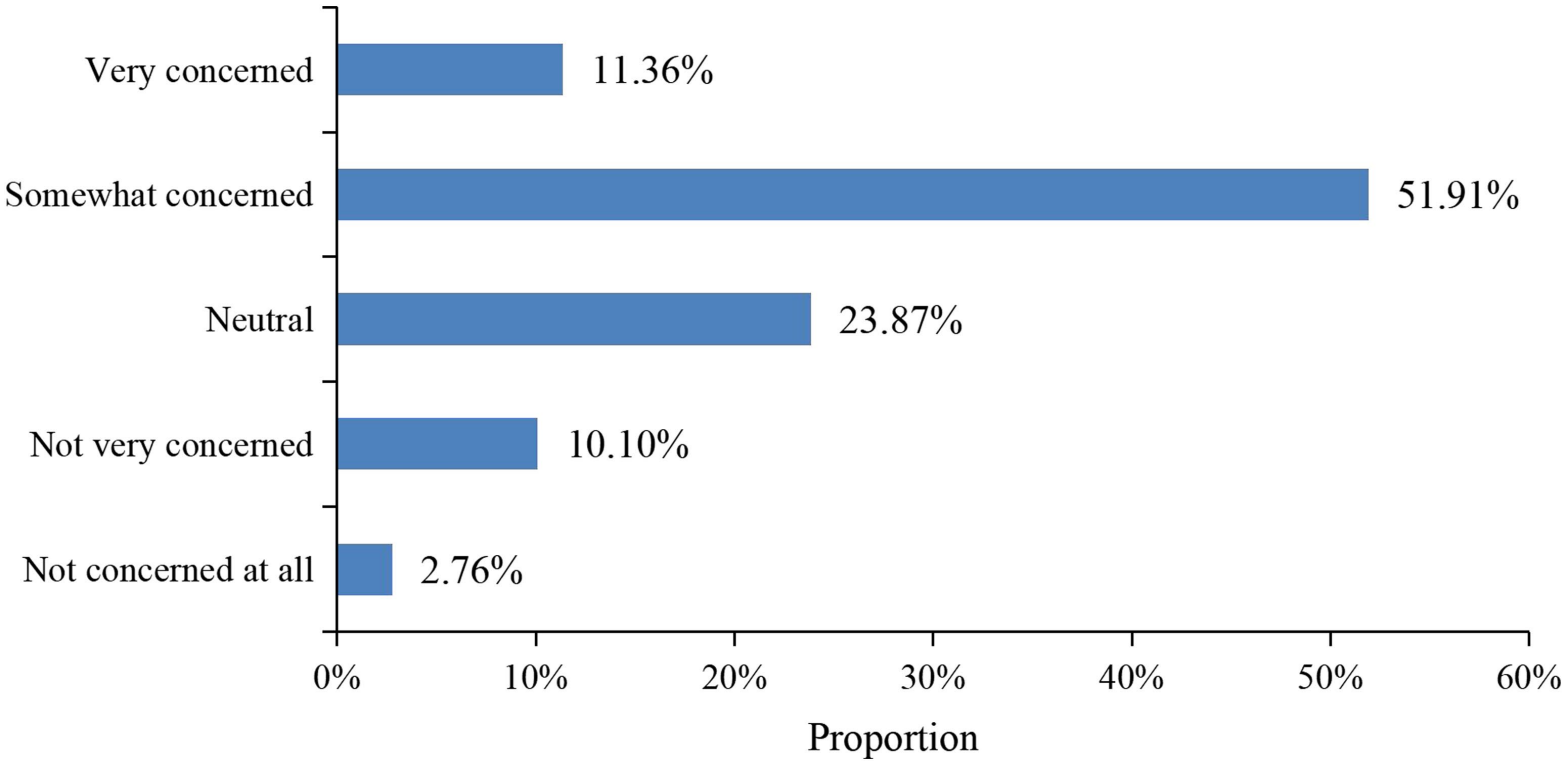
Distribution of public environmental concern.

In the survey of public environmental concern (see Figure 4), 51.91% of respondents reported being “somewhat concerned” about environmental issues, while only 11.36% were “very concerned.” The combined proportion of those who were “not unconcerned at all” and “not very concerned” was 12.86%. This suggests that although the public possesses a certain level of environmental awareness, the overall degree of concern still needs to be enhanced.

In summary, the public demonstrates a certain level of environmental concern and enthusiasm for pro-environmental behavior, but overall participation rates and the rate of translating concern into action remain relatively low. Moving forward, policy guidance and public education are needed to steer environmental awareness, further enhance the public’s pro-environmental behavioral engagement, and promote the popularization and deepening of pro-environmental behavior.

### 4.2 Benchmark regression

Before conducting the regression analysis, a multicollinearity test was performed. For the logistic regression model, the correlation coefficient matrix among the independent variables was calculated to assess multicollinearity, and the results showed low correlations between the variables. For the linear regression model, the variance inflation factor (VIF) for each variable was below 2.04, well below the threshold of 10, indicating no multicollinearity issue.

The regression analysis results are presented in Table 3. Columns (1) and (2) include only control variables; Columns (3) and (4) add the independent variable (experience of extreme weather events) to model; Columns (5) and (6) further include the mediating variable (public environmental concern) alongside the variables in Columns (3) and (4).

**Table 3:** Benchmark regression results.

Variable	(1)	(2)	(3)	(4)	(5)	(6)
	Public-sphere	Private-sphere	Public-sphere	Private-sphere	Public-sphere	Private-sphere
Age	−0.022^***^	−0.001	−0.022^***^	−0.001	−0.024^***^	−0.002
	(−0.006)	(−0.001)	(−0.006)	(−0.001)	(−0.006)	(−0.001)
Gender	0.142	0.009	0.142	0.008	0.156	0.010
	(−0.136)	(−0.038)	(−0.136)	(−0.038)	(−0.137)	(−0.037)
Marital status	−0.153	0.052	−0.160	0.049	−0.164	0.041
	(−0.182)	(−0.053)	(−0.181)	(−0.053)	(−0.184)	(−0.051)
Education level	−0.009	0.006	−0.010	0.006	−0.015	0.004
	(−0.021)	(−0.006)	(−0.021)	(−0.006)	(−0.022)	(−0.006)
Religion belief	−0.376	0.090	−0.435	0.083	−0.410	0.112
	(−0.523)	(−0.119)	(−0.531)	(−0.119)	(−0.528)	(−0.116)
Political affiliation	0.508^**^	0.010	0.535^***^	0.017	0.480^**^	−0.023
	(−0.199)	(−0.059)	(−0.200)	(−0.059)	(−0.201)	(−0.058)
Income level	0.003	0.003	0.004	0.003	0.005	0.003
	(−0.015)	(−0.004)	(−0.015)	(−0.004)	(−0.015)	(−0.004)
Household registration status	0.106	0.071	0.104	0.069	0.143	0.083^**^
	(−0.154)	(−0.043)	(−0.155)	(−0.043)	(−0.155)	(−0.042)
Socioeconomic status	0.167^*^	0.038	0.173^**^	0.039	0.151^*^	0.027
	(−0.088)	(−0.024)	(−0.088)	(−0.024)	(−0.088)	(−0.023)
Family economic status	0.027	0.006	0.035	0.008	0.028	−0.003
	(−0.103)	(−0.028)	(−0.103)	(−0.028)	(−0.103)	(−0.027)
Environmental self-efficacy	0.133^**^	0.025	0.134^**^	0.025	0.109^**^	0.012
	(−0.055)	(−0.016)	(−0.055)	(−0.016)	(−0.055)	(−0.015)
Experience with extreme weather events			0.131^**^	0.038^**^	0.120^**^	0.031^**^
			(−0.056)	(−0.015)	(−0.057)	(−0.015)
Environmental concern					0.336^***^	0.188^***^
					(−0.085)	(−0.020)
Province fixed effects	Yes	Yes	Yes	Yes	Yes	Yes
Constant	−2.162^**^	1.559^***^	−2.614^***^	1.427^***^	−3.672^***^	0.820^***^
	(−0.871)	(−0.198)	(−0.893)	(−0.204)	(−0.936)	(−0.209)
Observations	1929	1929	1929	1929	1925	1925
Adjusted *R*^2^		0.102		0.105		0.146

Standard errors are in parentheses; *p < 0.1, **p < 0.05, ***p < 0.01.

As shown in Table 3, the regression results from Models (3) and (4) indicate that experience with extreme weather events has a significant positive impact on both public-sphere and private-sphere pro-environmental behavior. Specifically, the regression coefficient for extreme weather experience on public-sphere pro-environmental behavior is 0.131 (*p* < 0.05), and on private-sphere pro-environmental behavior is 0.038 (p < 0.05). This suggests that the more severe the extreme weather experience, the higher the level of both public-sphere and private-sphere pro-environmental behavior. Thus, Hypothesis 1 is supported.

This result supports the claim that disaster experiences promote public pro-environmental attitudes or intentions [e.g., (24, 27, 46)]. However, since attitudes or intentions are ultimately different from actual behavior, this study focuses on the impact of extreme weather event experiences on public pro-environmental behavior, thereby taking one step further than those studies. Meanwhile, this result clearly contradicts existing findings that extreme weather events do not promote, or even negatively affect, public pro-environmental behavior (10, 11, 16, 26). A likely reason, as previously noted, is that studies using regional objective data on extreme weather events versus individual subjective experiences may yield different results. This is especially plausible given recent evidence of a “not-in-my-backyard” phenomenon: when people’s own postal area is directly hit by a flood, they are more likely to donate to environmental organizations and support green parties, but when a flood affects only neighboring areas, no such change occurs (9). Thus, it is reasonable to assume that such a discrepancy exists. Therefore, when examining the impact of extreme weather events on public pro-environmental behavior, it is essential to also consider the role of individual event experiences in order to fully capture the overall effect.

In Models (5) and (6), after including public environmental concern, experience with extreme weather events still has a significant positive effect on both public-sphere and private-sphere pro-environmental behavior, but the value of regression coefficients are reduced. Meanwhile, public environmental concern has a significant positive impact on public-sphere pro-environmental behavior (*β* = 0.336, *p* < 0.01), and on private-sphere pro-environmental behavior (*β* = 0.188, *p* < 0.01). This indicates that public environmental concern plays a mediating role in the relationship between extreme weather experience and the public’s pro-environmental behavior, providing necessary support for the subsequent mediation effect test.

### 4.3 Moderation effects

We examined the moderating role of media use on public-sphere and private-sphere pro-environmental behavior, with results shown in Tables 4, 5.

**Table 4:** Moderating effect results for public-sphere pro-environmental behavior.

Variable	(1)	(2)	(3)	(4)	(5)
	Public-sphere	Public-sphere	Public-sphere	Public-sphere	Public-sphere
Experience with extreme weather events	0.138^**^	0.140^**^	0.109^**^	0.110^**^	0.139^**^
	(0.056)	(0.056)	(0.054)	(0.055)	(0.057)
New media use	−0.037	−0.036			−0.017
	(0.063)	(0.063)			(0.063)
Experience with extreme weather events × new media use		−0.013			−0.016
		(0.038)			(0.039)
Traditional media use			0.318^***^	0.319^***^	0.319^***^
			(0.069)	(0.070)	(0.07)
Experience with extreme weather events × traditional media use				−0.005	−0.010
				(0.052)	(0.053)
Control variables	Yes	Yes	Yes	Yes	Yes
Province fixed effects	Yes	Yes	Yes	Yes	Yes
Constant	−1.607^**^	−1.613^**^	−0.698	−0.698	−1.097
	(0.768)	(0.769)	(0.457)	(0.457)	(0.783)

Standard errors are in parentheses; **p < 0.05, ***p < 0.01.

**Table 5:** Moderating effect results for private-sphere pro-environmental behavior.

Variable	(1)	(2)	(3)	(4)	(5)
	Private-sphere	Private-sphere	Private-sphere	Private-sphere	Private-sphere
Experience with extreme weather events	0.036^**^	0.036^**^	0.034^**^	0.033^**^	0.032^**^
	(0.015)	(0.015)	(0.015)	(0.015)	(0.015)
New media use	0.022	0.021			0.032^*^
	(0.017)	(0.017)			(0.017)
Experience with extreme weather events × new media use		−0.017^*^			−0.018^*^
		(0.010)			(0.0102)
Traditional media use			0.120^***^	0.121^***^	0.126^***^
			(0.020)	(0.020)	(0.020)
Experience with extreme weather events × traditional media use				0.016	0.013
				(0.015)	(0.015)
Control variables	Yes	Yes	Yes	Yes	Yes
Province fixed effects	Yes	Yes	Yes	Yes	Yes
Constant	1.510^***^	1.505^***^	1.767^***^	1.764***	1.729***
	(0.199)	(0.199)	(0.200)	(0.200)	(0.200)
Adjusted *R*^2^	0.106	0.108	0.122	0.122	0.125

Standard errors are in parentheses; *p < 0.1, **p < 0.05, ***p < 0.01.

The results in Table 4 show that new media use does not have a significant impact on public-sphere pro-environmental behavior. Moreover, the interaction term between new media use and extreme weather experience is also not significant. This indicates that new media use does not strengthen the effect of extreme weather experience on public-sphere pro-environmental behavior. On the other hand, as shown in Table 5, new media use does have a significant effect on private-sphere pro-environmental behavior. However, its interaction term with extreme weather experience is significantly negative. This suggests that new media use can weaken the positive impact of extreme weather experience on private-sphere pro-environmental behavior. Thus, Hypotheses 3a and 3b are not supported.

A possible explanation lies in the characteristics of public-sphere behaviors and the limitations of new media. Regarding the former, public-sphere behaviors (e.g., collective petitions) often require collective action and entail high costs in terms of time, social interaction, and risk, making their threshold much higher than that of private-sphere behaviors (e.g., saving electricity, waste sorting). Due to its convenience, new media more readily facilitates low-cost private-sphere behaviors. Regarding the latter, new media use inevitably exposes several problems (47). First, new media information is inherently fragmented and emotional, and algorithm-driven recommendation of homogeneous content can easily lead to information overload and burnout (48), resulting in “cognitive numbness” and avoidance, which may reduce action efficacy and fail to motivate high-threshold public-sphere behavior. Second, frequent use of new media may encourage low-cost online actions such as liking and sharing to replace offline participation, creating an illusion of “having contributed” (i.e., slacktivism) (49), thereby undermining motivation for real action. Third, the proliferation of conspiracy theories and misinformation on new media (50), coupled with generally low user trust in these platforms, can weaken the influence of information.

The results in Tables 4, 5 show that traditional media use has a significant positive impact on both public-sphere and private-sphere pro-environmental behavior. However, its interaction terms with extreme weather events experience are not significant for either public-sphere or private-sphere behavior. Therefore, Hypotheses 4a and 4b are not supported.

These results make sense given the nature of traditional media. Traditional media possess high authority and broad reach, allowing them to shape public pro-environmental behavior (51). Yet their drawbacks are clear: their information flow is largely one-way, lacking the interactivity of new media (52), which explains why their moderating role in individual disaster experiences is insignificant. Moreover, traditional media cover extreme weather events through a policy-oriented framework rather than personalized, emotional narratives (53, 54). This delivers highly homogeneous information to all individuals, regardless of personal experience, so the media’s effects differ little between groups. Furthermore, when traditional media clearly attribute extreme weather to climate change (33), individuals no longer need personal experience to perceive the issue as personally relevant. Nevertheless, the non-significant interaction between traditional media use and extreme weather experience does not imply traditional media are unimportant. Rather, it reveals a key feature: their influence on pro-environmental behavior is decontextualized and universally applicable (51). By consistently transmitting knowledge, shaping norms, and cultivating responsibility ethics, traditional media steadily enhance public pro-environmental behavior, irrespective of whether the public has personally experienced extreme weather.

### 4.4 Mediation effect analysis

This study examines the mediating role of public environmental concern in the relationship between experience of extreme weather events and the public’ pro-environmental behavior. Three methods were used for mediation analysis: stepwise regression, general structural equation modeling (GSEM), and the Karlson-Holm-Breen (KHB) method.

The stepwise regression results (see Table 6) show that the effect of extreme weather experience on public environmental concern is significant (*β* = 0.039, *p* < 0.05), indicating that extreme weather experience significantly enhance public environmental concern. After controlling for public environmental concern, the direct effect of extreme weather experience on public-sphere pro-environmental behavior remains positive and significant (*β* = 0.092, *p* < 0.1), while public environmental concern has a significant positive effect (*β* = 0.358, *p* < 0.01), indicating that public environmental concern plays a partial mediating role. This supports Hypothesis 2a. For private-sphere pro-environmental behavior, after controlling for public environmental concern, the direct effect of extreme weather experience on private-sphere pro-environmental behavior remains positive and significant (*α* = 0.035, *p* < 0.05), while public environmental concern has a significant positive effect (*β* = 0.185, *p* < 0.01), again indicating a partial mediating role. This supports Hypothesis 2b.

**Table 6:** Stepwise regression mediation effect analysis.

Variable	(1)	(2)	(3)	(4)	(5)
	Public environmental concern	Public-sphere	Public-sphere	Private-sphere	Private-sphere
Experience of extreme weather events	0.039^**^	0.102^*^	0.092^*^	0.042^***^	0.035^**^
Public environmental concern			0.358^***^		0.185^***^
Control variables	Yes	Yes	Yes	Yes	Yes
Constant	2.739^***^	−1.593^***^	−2.625^***^	1.761^***^	1.250^***^
Adjusted *R*^2^	0.035			0.031	0.072

*p < 0.1, **p < 0.05, ***p < 0.01.

Based on Hao et al. (55), we used the GSEM to analyze the mediation effect of public environmental concern. GSEM can handle various types of variables, including continuous, binary, categorical, and ordinal variables. It allows both linear and generalized linear response functions and is more tolerant of missing values (56). However, its limitations include the inability to provide standardized path coefficients or post-estimation model fit tests (56). In this study, public-sphere pro-environmental behavior is binary, private-sphere pro-environmental behavior is continuous, and both public environmental concern and extreme weather experience are ordinal variables. Therefore, using GSEM is appropriate. The results (see Table 7) show that public environmental concern plays an important mediating role between experience of extreme weather events and pro-environmental behavior.

**Table 7:** Mediation effect test based on GSEM.

Public-sphere pro-environmental behavior			Private-sphere pro-environmental behavior		
Direct effect	Indirect effect	Total effect	Direct effect	Indirect effect	Total effect
0.092^*^	0.014^**^	0.106^*^	0.035^**^	0.007^**^	0.042^***^

*p < 0.1, **p < 0.05, ***p < 0.01.

Following Malaju et al. (57), the KHB method was used for mediation effect analysis. The results are shown in Table 8. For public-sphere pro-environmental behavior, the direct effect of extreme weather events experience is 0.106 (*p* < 0.1), the total effect is 0.092 (*p* < 0.1), and the indirect effect (difference) is 0.014 (*p* < 0.05). For private-sphere pro-environmental behavior, the direct effect is 0.041 (*p* < 0.01), the total effect is 0.035 (*p* < 0.05), and the indirect effect is 0.007 (*p* < 0.05). These results indicate that public environmental concern plays a partial mediating role in both public- and private-sphere pro-environmental behavior, while the direct effect remains significant in both domains. This suggests that experience of extreme weather events not only indirectly influences pro-environmental behavior through public environmental concern but also directly promotes such behavior in both spheres.

**Table 8:** Mediation effect test based on KHB.

Public-sphere pro-environmental behavior			Private-sphere pro-environmental behavior		
Direct effect	Total effect	Effect difference	Direct effect	Total effect	Effect difference
0.106^*^	0.092^*^	0.014^**^	0.041^***^	0.035^**^	0.007^**^

*p < 0.1, **p < 0.05, ***p < 0.01.

## 5 Robustness checks

To ensure the robustness of the results, this study conducted robustness tests using three approaches. First, we employed the a fixed-effects model to control for unobservable individual heterogeneity. Second, we excluded specific variables, such as environmental self-efficacy, to assess their impact on the results. Third, we replaced key variables by assigning a value of 1 to respondents who did not answer the question “How severe do you think extreme weather events are in your residential area? (1 = not severe; 2 = not very severe; 3 = moderate; 4 = somewhat severe; 5 = very severe)” That is, non-response was interpreted as indicating that the respondent did not consider extreme weather to be severe. Overall, the main results remained stable across all three robustness tests (see Table 9), confirming the reliability of the findings.

**Table 9:** Robustness test results.

Variable	Fixed-effects model		Excluding specific variable		Replacing key variable	
	Public-sphere	Private-sphere	Public-sphere	Private-sphere	Public-sphere	Private-sphere
Experience of extreme weather events	0.136^**^	0.038^**^	0.146^***^	0.041^***^		
	(0.055)	(0.014)	(0.055)	(0.015)		
Proxy variable					0.129^***^	0.032^**^
					(0.049)	(0.013)
Environmental self-efficacy	0.112^**^	0.025			0.081	0.014
	(0.053)	(0.022)			(0.050)	(0.014)
Control variables	Yes	Yes	Yes	Yes	Yes	Yes
Constant		1.891^***^	−1.797^**^	1.474^***^	−2.004^***^	1.491^***^
		(0.144)	(0.768)	(0.195)	(0.757)	(0.187)
Adjusted *R*^2^		0.015		0.107		0.100

Standard errors are in parentheses; **p < 0.05, ***p < 0.01.

## 6 Addressing sample selection bias

Experience of extreme weather events has a significant positive impact on the public’s pro-environmental behavior. However, such experience may be influenced by individual characteristics and regional factors, which could also affect individuals’ pro-environmental behavior, potentially leading to sample selection bias. To address this issue, this study employs the propensity score matching (PSM) method. PSM matches individuals with extreme weather experience (treatment group) to those without (control group) based on their observed characteristics, achieving balance and comparability between the two groups and thereby allowing a valid comparison of pro-environmental behavior.

**Figure 5:**
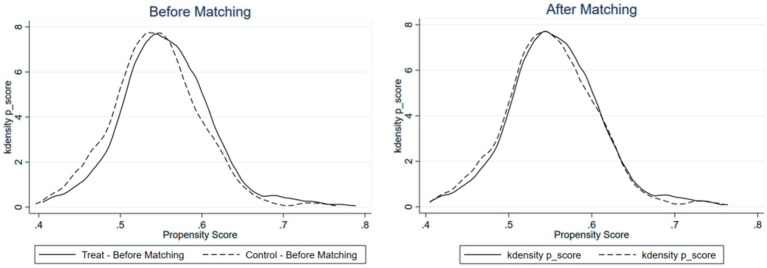
Kernel density function before and after matching.

In this study, the independent variable “experience of extreme weather events” was recoded as a binary dummy variable: samples with an experience level of 3 or higher were classified as having experienced extreme weather events (coded as 1), while those with a level below 3 were classified as not having experienced them (coded as 0). Figure 5 presents the kernel density functions of the treatment and control groups before and after PSM, intuitively showing the probability distribution of propensity scores in both groups. Before matching, clear differences existed between the two distributions. After matching, the distributions became more aligned, with a substantial reduction in differences. Moreover, most observations fell within the common support range, with only minimal sample loss. This satisfies the common support assumption. The kernel density plot shows that the matched distributions overlap more significantly, indicating a substantial reduction in propensity score differences between the treatment and control groups and thus a more balanced sample.

This study assesses post-matching balance using standardized differences and *t*-tests. The results (see Table 10) show that after matching, the absolute values of the standardized differences for all covariates between the treatment and control groups are less than 20%, indicating that the two groups are well balanced on these covariates. Furthermore, the *t*-test results show no significant differences in any covariate after matching, suggesting no systematic differences between the two groups. Therefore, the matched samples satisfy the balance assumption.

**Table 10:** Variable means and standard deviations test results before and after matching.

Variable	Sample	Mean		Standard deviation/%	Reduction in SD/%	*t*
		Treatment group	Control group			
Gender	Before matching	0.457	0.478	−4.6	97.0	−1.00
	After matching	0.457	0.456	0.1		0.03
Marital status	Before matching	0.765	0.796	−7.4	75.5	−1.62
	After matching	0.765	0.773	−1.8		−0.41
Education level	Before matching	10.609	9.356	15.5	96.4	3.38^***^
	After matching	10.053	10.027	0.6		0.13
Religion belief	Before matching	0.039	0.021	11.0	70.9	2.36^**^
	After matching	0.037	0.031	3.2		0.69
Income level	Before matching	9.264	8.943	6.9	90.4	1.52
	After matching	9.258	9.288	−0.7		−0.15
Household registration status	Before Matching	0.437	0.412	4.9	91.0	1.07
	After matching	0.437	0.435	0.4		0.10
Socioeconomic status	Before matching	2.251	2.288	−4.1	88.5	−0.89
	After matching	2.252	2.256	−0.5		−0.11
Family economic status	Before matching	2.595	2.609	−1.8	46.0	−0.39
	After matching	2.595	2.588	1.0		0.22
Environmental self-efficacy	Before matching	2.604	2.621	−1.5	75.0	−0.32
	After matching	2.607	2.611	−0.4		−0.08

Standard errors are in parentheses; **p < 0.05, ***p < 0.01.

This study employs the PSM method with three approaches (i.e., nearest neighbor matching, kernel matching, and caliper matching) to mitigate potential sample selection bias. The results are presented in Table 11. The estimated average treatment effect on the treated (ATT) from all three matching methods is positive and significant at the 10% level or better, indicating that extreme weather experience has a significant positive effect on the public’s pro-environmental behavior. This further supports the findings of the baseline regression analysis.

**Table 11:** Estimation results of average treatment effect.

Matching method	Public-sphere pro-environmental behavior		Private-sphere pro-environmental behavior	
	ATT	*t*	ATT	*t*
Nearest neighbor matching	0.032	1.82^*^	0.084	2.06^**^
Kernel matching	0.272	1.67^*^	0.086	2.28^**^
Caliper matching	0.032	1.82^*^	0.083	2.04^**^

*p < 0.1, **p < 0.05.

## 7 Heterogeneity analysis

This study examines the heterogeneous effects of experience of extreme weather events on public-sphere and private-sphere pro-environmental behaviors across different groups (Southern vs. Northern, rural vs. non-rural, high vs. low environmental self-efficacy). The Chow test is employed to examine the differences in coefficients between groups, effectively assessing whether the impact of the independent variable varies significantly across subgroups.

Significant differences exist between southern and northern China in terms of climate conditions, economic development, and cultural backgrounds. Southern regions typically face more extreme precipitation and heatwave events, while northern regions are more affected by droughts and extreme cold. These differences may lead to variations in residents’ perceptions of climate disasters and their subsequent pro-environmental behaviors. The regional heterogeneity analysis (Table 12) shows that extreme weather events experience has a significantly positive impact on public-sphere pro-environmental behaviors of citizens in the South, whereas its impact on citizens in the North is not significant. For private-sphere pro-environmental behavior, the impact is significantly positive in the South but smaller and non-significant in the North. The coefficient difference test indicates that the regional difference in private-sphere pro-environmental behaviors is significant (*p* = 0.044), while the difference in public-sphere pro-environmental behaviors is not significant (*p* = 0.578).

**Table 12:** Results of regional heterogeneity analysis.

Variable	Public-sphere		Private-sphere	
	South	North	South	North
Experience of extreme weather events	0.146^*^	0.096	0.065^***^	0.011
	(0.075)	(0.084)	(0.020)	(0.024)
Control variables	Yes	Yes	Yes	Yes
Province fixed effects	Yes	Yes	Yes	Yes
Constant	−2.055^***^	−1.336^*^	1.852^***^	1.598^***^
	(0.619)	(0.787)	(0.172)	(0.229)
Observations	1,105	824	1,105	824
Adjusted *R*^2^			0.030	0.060
*p*-value of effect difference	0.578		0.044	

Standard errors are in parentheses; *p < 0.1, ***p < 0.01.

A possible explanation for these results is that subjective attribution plays an important role in the relationship between extreme weather experiences and public pro-environmental behavior (58, 59), and that such attribution varies significantly across regions as it is shaped by individuals’ psychological and social context (60). In southern China, where extreme weather events such as typhoons, heavy rainfall, and floods are common, residents’ attributional reasoning tends to be more explicit and direct. This is driven by two logics. First, governments and media in the South more frequently link extreme weather to climate change in disaster coverage. Second, frequent and intense disaster experiences reduce the likelihood of attributing events to mere “natural fluctuations.” Once attribution is clear, individuals who have experienced extreme weather are more likely to interpret it as “caused by human activity,” thereby activating self-identity-driven changes in private-sphere pro-environmental behavior.

In contrast, northern China experiences extreme weather events such as cold waves and dust storms, which are often perceived as part of “natural patterns.” Given the relatively lower frequency and intensity of disasters in the North, such ambiguous attribution fails to generate the psychological drive of “I need to change.” Meanwhile, public-sphere pro-environmental behavior depends more on social norms and knowledge levels (17, 61), which may not differ substantially between southern and northern residents. The dissemination of social norms relies primarily on media and policy campaigns, and the distribution of environmental knowledge and institutional trust in the public-sphere is relatively balanced across China. Therefore, no significant regional difference exists in public-sphere pro-environmental behavior.

Rural areas lag behind in infrastructure, information access, and economic resources, potentially shaping residents’ awareness and adaptive capacity to climate disasters. Table 13 shows that experience of extreme weather events has a significantly positive impact on public-sphere pro-environmental behaviors in non-rural/urban areas, but a non-significant effect in rural areas. For private-sphere behavior, the pattern is similar: significant in non-rural/urban areas, non-significant in rural areas. The coefficient difference test indicates that the urban–rural gap in public-sphere pro-environmental behaviors is marginally significant (*p* = 0.057), while the gap in private-sphere pro-environmental behaviors is not (*p* = 0.433).

**Table 13:** Results of urban–rural heterogeneity analysis.

Variable	Public-sphere		Private-sphere	
	Non-rural	Rural	Non-rural	Rural
Experience of extreme weather events	0.248^***^	0.051	0.052^**^	0.032
	(0.092)	(0.074)	(0.024)	(0.020)
Control variables	Yes	Yes	Yes	Yes
Province fixed effects	Yes	Yes	Yes	Yes
Constant	−0.479	−2.995^***^	1.850^***^	1.328^***^
	(0.965)	(1.042)	(0.488)	(0.251)
Observations	821	1,108	821	1,108
Adjusted *R*^2^			0.154	0.084
*p*-value of effect difference	0.057		0.433	

Standard errors are in parentheses; **p < 0.05, ***p < 0.01.

The above results are also interpretable. Urban residents have more frequent exposure to climate-change-related news reports, social media discussions, and community activities, which strengthens their environmental concern and reduces their tendency to believe in climate change conspiracy theories (62), thereby shortening the psychological distance of this global issue (22). Consequently, when extreme weather occurs, urban residents are more likely to establish a clear causal link between the disaster and climate change, triggering motivation for public-sphere action. In contrast, rural residents tend to attribute extreme weather to “natural patterns,” “acts of nature,” or “local environmental changes” rather than to abstract global climate change. Such local attribution, closely related to their knowledge gap (63), struggles to generate the motivation needed for collective climate action.

Regarding the finding of no significant urban–rural difference in private-sphere behavior, a possible explanation is that many private-sphere pro-environmental actions (e.g., saving water and electricity, reducing waste, and reusing old items) are inherent parts of rural livelihood culture (64). In rural communities, resource conservation stems less from “environmental awareness” and more from economic pressures and intergenerationally transmitted habits. Such habitual private-sphere behaviors already exist without needing to be “triggered” by extreme weather; therefore, no statistically significant additional increase occurs after a disaster.

Environmental self-efficacy refers to an individual’s belief that their own actions can effectively improve environmental problems. Individuals with high environmental self-efficacy are more likely to engage in pro-environmental behaviors, as they believe their efforts make a positive difference. In the heterogeneity analysis based on environmental self-efficacy (Table 14), extreme weather experience has a significantly positive impact on public-sphere pro-environmental behaviors among those with high environmental self-efficacy, but no significant impact among those with low environmental self-efficacy. For private-sphere pro-environmental behaviors, the effect is significantly positive for the high-efficacy group, yet non-significant for the low-efficacy group. The coefficient difference test shows that the gap in public-sphere pro-environmental behaviors between the two groups is marginally significant (*p* = 0.082), while the gap in private-sphere behaviors is not (*p* = 0.818).

**Table 14:** Results of environmental self-efficacy heterogeneity analysis.

Variable	Public-sphere		Private-sphere	
	High efficacy	Low efficacy	High efficacy	Low efficacy
Experience of extreme weather events	0.125^**^	0.043	0.037^**^	0.052
	(0.062)	(0.149)	(0.016)	(0.043)
Control variables	Yes	Yes	Yes	Yes
Province fixed effects	Yes	Yes	Yes	Yes
Constant	−2.620^**^	−1.224	1.622^***^	0.972^*^
	(1.149)	(1.765)	(0.217)	(0.541)
Observations	1,635	294	1,625	294
Adjusted *R*^2^			0.112	0.153
*p*-value of effect difference	0.082		0.818	

Standard errors are in parentheses; *p < 0.1, **p < 0.05, ***p < 0.01.

The reasons for the above results are quite clear. Private-sphere pro-environmental behaviors face fewer structural barriers and are primarily driven by personal agency and self-efficacy (17), making them generally easier to adopt than public-sphere behaviors. In contrast, public-sphere pro-environmental behaviors typically entail higher social participation costs, stronger collective action coordination capacity, and a belief in the effectiveness of such actions. Only individuals with high environmental self-efficacy possess the psychological capital to translate risk perception into public-sphere action as they believe they can make a difference through collective efforts and are thus willing to engage in public participation. Individuals with low environmental self-efficacy, however, are discouraged by doubts about their own abilities or the effectiveness of collective action.

Regarding the finding of no significant difference in private-sphere pro-environmental behavior, a possible explanation is that many private-sphere behaviors are less dependent on environmental self-efficacy. A considerable proportion of these behaviors are habitual, already embedded in the routine patterns of daily life.

Overall, the impact of extreme weather experience on public and private pro-environmental behaviors exhibits certain heterogeneity across regions, urban–rural areas, and levels of environmental self-efficacy. The effects are more pronounced in the South, non-rural areas, and among individuals with high environmental self-efficacy, while they are weaker in the North, rural areas, and among those with low environmental self-efficacy. This suggests that when formulating relevant policies, it is necessary to account for regional, urban–rural, and group differences to enhance policy targeting and effectiveness.

## 8 Conclusions and discussion

### 8.1 Conclusion

Using data from the 2021 Chinese General Social Survey (CGSS), this study empirically examines the impact of extreme weather events experience on the public’s pro-environmental behavior, along with the moderating effects of media use and the mediating role of public environmental concern. The main findings are as follows.

First, experience of extreme weather events significantly and positively affects both public- and private-sphere pro-environmental behaviors, that is, more severe disaster experiences lead to greater pro-environmental action in both spheres. Second, public environmental concern partially mediates the relationship, such that extreme weather experience indirectly promotes pro-environmental behavior by enhancing public environmental concern. Third, new media use, however, negatively moderates the effect for private-sphere behavior, weakening the direct influence of extreme weather events. Finally, significant heterogeneity exists across regions, urban–rural areas, and levels of environmental self-efficacy. Residents in the South, non-rural areas, and those with high environmental self-efficacy are more responsive to extreme weather disasters, while their counterparts in the North, rural areas, and with low efficacy show weaker responses.

### 8.2 Policy implications

Based on the above findings, several policy implications are proposed.

First, leverage extreme weather events as catalysts for pro-environmental behavior. Given that more severe extreme weather events experiences significantly increase both public- and private-sphere pro-environmental behaviors, governments and relevant agencies should systematically integrate behavior-promoting initiatives (e.g., resource conservation, low-carbon transport, community environmental projects) into post-disaster recovery and emergency management, thereby reinforcing the positive pathway from disaster experience to action.

Second, strengthen public environmental concern as a mediating mechanism. Since extreme weather experience indirectly promotes pro-environmental behavior by enhancing public environmental concern, authorities should improve environmental risk communication and education, explicitly linking extreme weather events to long-term issues such as climate change and ecological protection. Schools, communities, and traditional media should be leveraged to raise public awareness and sense of responsibility toward environmental issues.

Third, optimize new media communication to better guide private-sphere behavior. In response to the finding that new media use negatively moderates the effect of extreme weather experience on private-sphere pro-environmental behavior, media platforms should reduce fragmented, disaster-sensationalizing content. Instead, they should provide clear, concrete, and actionable guidance for private-sphere behaviors (e.g., household energy saving, waste sorting, green consumption) to avoid information overload or emotional fatigue that may suppress action.

Finally, adopt differentiated policies to support less responsive groups and regions. Given that residents in the South, non-rural/urban areas, and those with high environmental self-efficacy respond more strongly to extreme weather, while their counterparts in the North, rural areas, and with low environmental self-efficacy show weaker responses, differentiated intervention strategies are recommended. For less responsive regions and groups, policies should enhance infrastructure resilience, provide community-based environmental skills training, and implement psychological empowerment programs to boost environmental self-efficacy, thereby narrowing the behavioral response gap.

## Data Availability

The data in the study are publicly available on the CGSS official website (http://cgss.ruc.edu.cn/).
